# Poly-Tobacco Use among High School Students

**DOI:** 10.3390/ijerph121114477

**Published:** 2015-11-13

**Authors:** Sarah D. Kowitt, Tanha Patel, Leah M. Ranney, Li-Ling Huang, Erin L. Sutfin, Adam O. Goldstein

**Affiliations:** 1 Department of Health Behavior, Gillings School of Global Public Health, University of North Carolina at Chapel Hill, Chapel Hill, NC 27599, USA; 2 North Carolina Department of Health and Human Services, Tobacco Prevention and Control Branch, Raleigh, NC 27609, USA; E-Mail: tanha2101@gmail.com; 3 Department of Family Medicine, University of North Carolina at Chapel Hill, Chapel Hill, NC 27599, USA; E-Mails: Leah_Ranney@unc.edu (L.M.R.); adam_goldstein@med.unc.edu (A.O.G); 4 Lineberger Comprehensive Cancer Center, University of North Carolina at Chapel Hill, Chapel Hill, NC 27599, USA; E-Mail: liling_huang@med.unc.edu; 5 Department of Social Sciences and Health Policy, Wake Forest School of Medicine, Winston-Salem, NC 27157, USA; E-Mail: esutfin@wakehealth.edu

**Keywords:** tobacco products, non-cigarette tobacco products, adolescents, health beliefs, tobacco policy

## Abstract

Although cigarette use by adolescents is declining, emerging tobacco products are becoming increasingly popular and youth may use more than one type of tobacco product. The purposes of this study were: (1) to assess patterns of poly-tobacco use among a representative sample of high school students and (2) to determine how beliefs correlate with poly-tobacco use. Data came from the 2013 North Carolina Youth Tobacco Survey (*n* = 4092). SAS logistic regression survey procedures were used to account for the complex survey design and sampling weights. Among all high school students in NC in 2013, 29.7% reported current any tobacco use, with 19.1% reporting current poly-tobacco use, and 10.6% reporting current use of only one product. Among poly-tobacco users, 59.3% reported that one of the products they currently used was cigarettes. Positive tobacco product beliefs were found to be significantly associated with poly-tobacco use. Communication campaigns, policy efforts, and future research are needed for prevention, regulation, and control of poly-tobacco use among adolescents, which represents a significant public health problem.

## 1. Introduction

The substantial decline in adolescent cigarette use over the past few decades is a notable public health achievement [1]. However, this decline is threatened by a recent increase in use of emerging tobacco products, such as e-cigarettes, hookah, and snus. In a 2012 national study, 71.6% of high school students were aware, 21.7% had ever used, and 8.8% had currently used one or more emerging products in the past month [2]. These prevalence rates, particularly for e-cigarettes [3] and hookah [4], were considerably higher than in previous years.

Evidence exists that adolescents often use more than one tobacco product. A web-based survey of 448 adolescents aged 16–24 in 2012 and 2013 found that among current users of tobacco, the prevalence of dual use (defined as use of two tobacco products in the past 30 days) was 25% and the prevalence of multiple use (defined as use of three or more tobacco products in the past 30 days) was 21% [5]. Moreover, among those currently using tobacco products, almost equal numbers of adolescents used two or more products (46%) compared to those exclusively using one product (54%) [5]. Similar findings were reported in a nationally representative study of middle and high school students in 2012, where use of tobacco products other than cigarettes was more prevalent than exclusive use of cigarettes [6]. Most respondents who used tobacco products used two products (prevalence of 5.4% among those aged 15–17) or three or more products (prevalence of 6.0% among those aged 15–17) [6]. In the most recent version of the National Youth Tobacco Survey (NYTS), the prevalence of two or more tobacco products was 12.7% in 2014 [7]. The high prevalence of dual and poly-tobacco use is noteworthy given that these products may produce dependence and have toxic effects for adolescents’ developing brains [8].

Established theories on adolescent tobacco use suggest that beliefs, such as perceived susceptibility, severity, barriers, and benefits—all of which are found in the Health Belief Model, are influential in predicting smoking intentions and behaviors [9,10,11,12]. Despite existing studies on the prevalence of multiple tobacco use, only one study, to our knowledge, has examined how beliefs may correlate with dual and poly-tobacco use of conventional and emerging tobacco products among adolescents. This study found that adolescents who believed that breathing smoke from tobacco products caused harm were less likely to be poly-tobacco users, but there was no association between dual and poly-tobacco product use and agreement with the statement “all tobacco products are dangerous” [6]. Moreover, no other studies have examined adolescents from a major tobacco growing state (including Kentucky, North Carolina, South Carolina, Tennessee, and Virginia) where there is reason to believe that tobacco patterns among youth may differ [13]. The purposes of this study were: (1) to assess patterns of poly-tobacco use among a representative sample of high school students and (2) to determine how beliefs correlate with poly-tobacco use.

## 2. Experimental Section

### 2.1. Questionnaire

Data utilized in this research come from the 2013 North Carolina Youth Tobacco Survey (NCYTS). The NCYTS is a public and charter school-based survey of students in grades 6–12. For the purposes of this study, we restricted analyses to students in grades 9–12. Administered by the Tobacco Prevention and Control Branch (TPCB) of the North Carolina Department of Health and Human Services, the NCYTS has been administered every two years since 1999. Similar to the NYTS [14], the purpose of the NCYTS is to provide data on long-term, intermediate, and short-term indicators for the design, implementation, and evaluation of state tobacco prevention and control programs. The survey measures students’ tobacco-related beliefs and behaviors.

A total of 68 questions were asked in the 2013 NCYTS. Among the 68 questions, approximately 53 were core questions, which are questions used by all states administering a youth tobacco survey (YTS) and the NYTS. The core questions are asked with each survey administration to compare the prevalence of tobacco use at the national-level. Seven questions in the 2013 NCYTS addressed students’ beliefs about cigarettes and/or tobacco products (Supplementary Table S1). These questions assessed students’ opinions of tobacco companies, harmfulness of breathing smoke from tobacco products, coolness of smoking, popularity of smokers, dangerousness of tobacco products, and susceptibility to smoking.

Opinions of tobacco companies was measured with the question, “Do you believe that tobacco companies try to get young people under 18 to use tobacco products?” with response options for “Yes” and “No”. Harmfulness of breathing smoke from tobacco products was measured with the question, “Do you think that breathing smoke from other people’s cigarettes or other tobacco products is...?” with response options dichotomized for “Very or somewhat harmful to one’s health” and “Not very or not harmful at all to one’s health”. Coolness of smoking was measured with the question, “Do you think smoking cigarettes makes young people look cool or fit in?” with response options dichotomized for “Definitely or probably yes” and “Definitely or probably not”. Popularity of smokers was measured with the question, “Do you think young people who smoke cigarettes have more friends?” with response options dichotomized for “Definitely or probably yes” and “Definitely or probably not”. Dangerousness of tobacco products was measured with the question, “How strongly do you agree with the statement ‘All tobacco products are dangerous’?” with response options dichotomized for “Strongly agree or agree” and “Strongly disagree or disagree”. Perceived susceptibility to smoking was measured with two questions, “Do you think you will smoke a cigarette in the next year?” and “If one of your best friends were to offer you a cigarette, would you smoke it?”. Response options were dichotomized for “Definitely or probably yes” and “Definitely or probably not”.

The dependent variable of interest, current poly-tobacco use, was defined as use of two or more tobacco products in the past 30 days, including: cigarettes; chewing tobacco, snuff, or dip; cigars, cigarillos, or little cigars; tobacco in a pipe; bidis; clove cigarettes; roll-your-own cigarettes; flavored cigarettes; clove cigars; hookah or waterpipe; flavored little cigars; snus; dissolvable tobacco products; e-cigarettes; and any other new product not listed above. Single use was classified as current use of only one of the products listed above. Non-use was defined as not using any of the products listed above. Current use of tobacco products was defined as use of a product on at least one day of the past 30 days. Control variables included sex (dichotomized as female or male), age (treated as a continuous variable), and race/ethnicity (treated as categorical with variables for White, Black, Hispanic, and other).

### 2.2. Sampling

The sampling frame for this study was the 2013 NCYTS of North Carolina (NC) high school (9th–12th grade) students. A multi-stage cluster sample design in three distinct regions of the state (west, central, and east) was used, with Local Education Area (school district) serving as the primary sampling unit and school serving as the secondary sampling unit. Classes were randomly selected within each school, excluding special populations (e.g., special needs, English as Second Language). Since the beginning of NCYTS (1999), survey statisticians have used a two-stage cluster sampling method in which school districts were first selected within three geographic regions of the state. A school’s probability for selection was proportional to its enrollment size for the survey year. During the second stage, second period classes were randomly selected within the selected schools and all the students within a selected class were eligible to participate in the survey except for those who were exempt from other written tests due to language or learning barriers. Participation was voluntary and anonymous. Passive consent forms were utilized, unless an active consent form was required according to a specific school district policy. Students recorded their responses on scannable sheets, which were then returned to the Tobacco Prevention and Control Branch for processing. The completed answer sheets were cleaned and analyzed by RTI International and the CDC before being released to the TPCB.

### 2.3. Analysis

We used SAS version 9.3 survey procedures (SAS Inc., Cary, NC, USA) to account for the complex survey design and sampling weights [15]. All analyses were conducted between May 2014 and February 2015. Descriptive analyses and cross-tabulations were used to generate weighted percentages and confidence intervals of independent and dependent variables. We entered all independent variables simultaneously in the logistic regression model to identify variables that were significantly related to multiple tobacco use. A multivariate ordinal logistic regression model with cumulative logits was used to examine the association between relevant predictors and use of tobacco products at three levels: non-use (*i.e.*, not using any tobacco products in the past 30 days), single use (*i.e.*, use of one tobacco product in the past 30 days), and poly-use (*i.e.*, use of two or more tobacco products in the past 30 days) [16]. The odds of outcomes were cumulated over the lowest order category, *i.e.*, non-use. Results include weighted percentages, adjusted odds ratios (AOR), and confidence intervals (CI) and may be generalized to all North Carolina high school students attending public or charter schools. For all analyses, significance was set at *p* < 0.05.

Only individuals with complete data across all relevant variables were included in the analyses. However, this approach (*i.e.*, listwise deletion) for handling missing data may produce biased results if cases are not missing at random. As a result, we used multiple imputation to impute missing data as a sensitivity analysis [17]. Twenty multiply-imputed complete data sets were created using SAS Proc MI [15]. Each imputed data set was analyzed in the logistic regression model described above and combined using Proc MIAnalyze [15] to determine if use of multiple imputation produced different results than listwise deletion.

## 3. Results and Discussion

### 3.1. Results

In 2013, among students attending 83 participating high schools (out of 102 schools sampled), 4092 high school students (out of 4908 sampled) responded resulting in an overall response rate of 67.8%. Data needed to compute analyses were missing from 0.1% to 3.0%. In the final logistic regression model, 217 observations were deleted (*i.e.*, 5.3% of all observations). Analyzing the data with multiple imputation did not change our conclusions; all significant parameters remained significant.

Table 1 provides participants’ demographic information as well as weighted percentages for control and predictor variables included in the ordinal logistic regression model. Data are provided for all students and differentiated by the status of tobacco use, *i.e.*, non-users, single-users, and poly-users. In 2013, the sample was approximately half female (48.9%); mostly between the ages of 14 years and 17 years; and approximately 54.0% non-Hispanic White, 27.3% non-Hispanic Black, 11.2% Hispanic, and 7.5% non-Hispanic Other.

**Table 1:** Weighted percentages for independent variables used in the ordinal logistic regression model for the 2013 North Carolina Youth Tobacco Survey.

Variable	All 2013 High School Students, % (*n* = 4092) ^a^	Non-Users, % (*n* = 2857)	Single-Users, % (*n* = 417)	Poly-Users, % (*n* = 751)
Gender				
Female	48.9	54.2	41.1	34.4
Male	51.1	45.8	58.9	65.6
Age ^b^				
14 years	19.6	22.7	14.7	11.1
15 years	25.2	27.3	20.4	20.1
16 years	24.4	24.1	27.9	23.6
17 years	23.1	20.1	27.5	32.4
18 years	6.6	5.2	8.7	10.2
19 years or older	0.4	0.2	0.7	1.1
Race				
Non-Hispanic White	54.0	52.4	50.5	63.2
Non-Hispanic Black	27.3	29.0	30.3	19.1
Hispanic	11.2	11.6	9.7	10.4
Non-Hispanic other	7.5	7.0	9.5	7.3
Believe tobacco companies try to get young people to use tobacco products				
Yes	58.0	61.8	51.1	48.5
No	42.0	38.2	48.9	51.5
Believe that breathing smoke from other people’s cigarettes or other tobacco products is				
Very or somewhat harmful to one’s health	89.6	93.2	91.3	75.1
Not very or not harmful to one’s health	10.4	6.8	8.7	24.9
Think that smoking cigarettes makes young people look cool or fit in				
Definitely yes or probably yes	12.8	7.7	18.7	28.0
Definitely not or probably not	87.2	92.3	81.3	72.0
Think that young people who smoke cigarettes have more friends				
Definitely yes or probably yes	24.5	21.2	29.9	33.2
Definitely not or probably not	75.5	78.8	70.1	66.8
Believe that “All tobacco products are dangerous”				
Strongly agree or agree	85.9	91.4	81.5	68.8
Strongly disagree or disagree	14.1	8.6	18.5	31.2
Will smoke a cigarette in the next year				
Definitely yes or probably yes	15.9	2.9	24.0	57.9
Definitely no or probably no	84.1	97.1	76.0	42.1
Would smoke a cigarette offered by a best friend				
Definitely yes or probably yes	16.8	3.6	24.3	59.3
Definitely no or probably no	83.2	96.4	75.7	40.7

**^a^** All estimates were calculated using listwise deletion. ^b^ Age is treated as a continuous variable in the logistic regression model.

Overall, 29.7% (95% CI: 27.1–32.3) of high school students reported current use of any tobacco product, and 69.8% (95% CI: 67.3–72.4) did not use any tobacco product in the past 30 days. The prevalence of poly-tobacco use among high school students was 19.1% (95% CI: 17.3–20.8; *n* = 751). The prevalence of single use among high school students was 10.6% (95% CI: 9.3–11.9; *n* = 417) in 2013. The prevalence of poly-tobacco use was almost twice as large as the prevalence of single tobacco use.

Among poly-tobacco users, the most common products used (in addition to another product) were: cigarettes (59.3%, 95% CI: 54.9–63.7), followed by cigars/cigarillos/little cigars (54.3%, 95% CI: 50.6–58.0), chewing tobacco, snuff, or dip (35.0%, 95% CI: 29.9–40.1), tobacco in a pipe (34.8%, 95% CI: 31.4–38.1), and e-cigarettes (32.0%, 95% CI: 26.1–37.9). Products used the least by poly-users included bidis (11.5%, 95% CI: 8.3–14.7), clove cigars (8.8%, 95% CI: 6.9–10.8), and dissolvable tobacco products (3.7%, 95% CI: 2.2–5.2).

**Figure 1:**
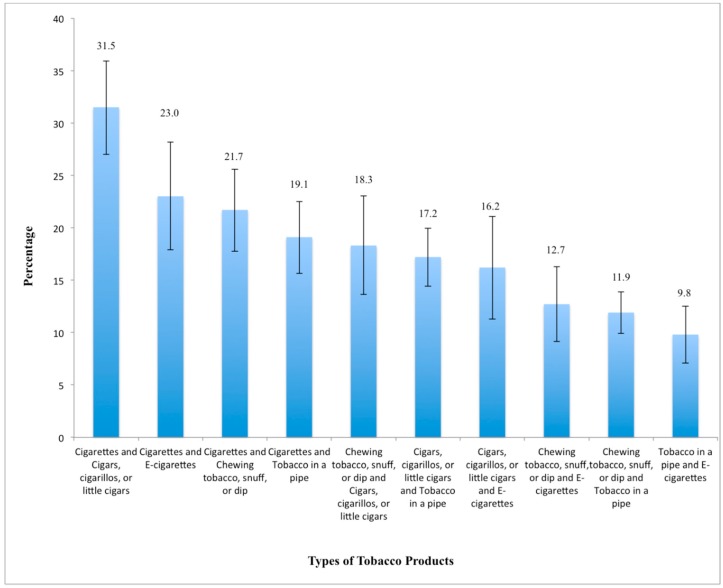
Combinations of Tobacco Products among poly-users, 2013 North Carolina Youth Tobacco Survey. Percentages do not add up to 100% because students may use more than one tobacco product, the denominator is *n* = 751 (*i.e.*, poly-tobacco users).

The most common combination of products used by poly-tobacco users was cigarettes and cigars/cigarillos/or little cigars (31.5%, 95% CI: 27.0–35.9), followed by cigarettes and e-cigarettes (23.0%, 95% CI: 17.9%–28.2%) (Figure 1).

Table 2 shows results from the multivariable ordinal logistic regression analysis conducted using the 2013 data. As student age increased, students were significantly more likely to report greater use of tobacco products, both for poly-use *versus* single or non-use and for poly or single-use *versus* non-use (AOR, 1.3; 95% CI, 1.2–1.6). Compared to female students, male students were significantly more likely to report greater use of tobacco products, both for poly-use *versus* single or non-use and for poly or single-use *versus* non-use (AOR, 2.1; 95% CI, 1.4–3.0).

There was no association with use of tobacco products by race for White students (AOR, 1.1; 95% CI, 0.9–1.3), Hispanic students (AOR, 0.8; 95% CI, 0.6–1.1) or non-Hispanic other students (AOR, 1.0; 95% CI, 0.5–1.7) compared to Black students. Thus, overall, students reporting greater use of tobacco products were more likely to be older and male both for poly-use *versus* single or non-use and for poly or single-use *versus* non-use.

**Table 2:** Weighted ordinal logistic regression results for the 2013 North Carolina Youth Tobacco Survey.

Variable	Use of Tobacco Products ^b,c^ Adjusted Odds Ratio (95% CI)
Gender	
Female	Ref.
Male	2.1 ^a^ (1.4–3.0)
Age ^d^	1.3 ^a^ (1.2–1.5)
Race	
Non-Hispanic Black	Ref.
Non-Hispanic White	1.1 (0.9–1.3)
Non-Hispanic other	1.0 (0.5–1.7)
Hispanic	0.8 (0.6–1.1)
Believe tobacco companies are trying to get young people to use tobacco products	
Yes	Ref.
No	1.3 ^a^ (1.2–1.6)
Believe that breathing smoke from other people’s cigarettes or other tobacco products is	
Very or somewhat harmful to one’s health	Ref.
Not very or not harmful to one’s health	1.6 ^a^ (1.2–2.2)
Think that smoking cigarettes makes young people look cool or fit in	
Definitely not or probably not	Ref.
Definitely yes or probably yes	2.0 ^a^ (1.3–3.2)
Think that young people who smoke cigarettes have more friends	
Definitely not or probably not	Ref.
Definitely yes or probably yes	1.2 (0.9–1.6)
Believe that “All tobacco products are dangerous”	
Strongly agree or agree	Ref.
Strongly disagree or disagree	3.2 ^a^ (2.5–4.2)
Will smoke a cigarette in the next year	
Definitely yes or probably yes	Ref.
Definitely no or probably no	6.3 ^a^ (4.3–9.2)
Would smoke a cigarette offered by a best friend	
Definitely yes or probably yes	Ref.
Definitely no or probably no	4.9 ^a^ (3.5–6.8)

Abbreviation: CI, Confidence Interval; Ref, reference group. ^a^ Statistically significant at the *p* < 0.05 level. **^b^** Use of tobacco products is modeled in an ordinal logistic regression model with categories for: poly-tobacco use (*n* = 751), single-use (*n* = 417), and non-use (*n* = 2857). **^c^** All estimates were calculated by using listwise deletion. ^d^ Age is treated as a continuous variable in the logistic regression model.

High school students who endorsed more positive beliefs about tobacco companies and tobacco products were more likely to report greater use of tobacco products, both for poly-use *versus* single or non-use and for poly or single-use *versus* non-use. Specifically, greater use was significantly associated with students believing that tobacco companies are not trying to get young people to use tobacco products (AOR, 1.3; 95% CI, 1.2–1.6), believing that breathing smoke from other people’s cigarettes or other tobacco products is not very or not harmful to one’s health (AOR, 1.6; 95% CI, 1.2–2.2), thinking that smoking cigarettes makes young people look cool or fit in (AOR, 2.0; 95% CI, 1.3–3.2), strongly disagreeing or disagreeing that all tobacco products are dangerous (AOR, 3.2; 95% CI, 2.5–4.2), believing that they will definitely or probably smoke a cigarette in the next year (AOR, 6.3; 95% CI, 4.3–9.2), and believing that they would definitely or probably smoke a cigarette offered by a best friend (AOR, 4.9; 95% CI, 3.5–6.8). There was no association between believing that young people who smoke cigarettes definitely or probably have more friends and greater use of tobacco products (AOR, 1.2; 95% CI, 0.9–1.6). Thus, overall, students with positive beliefs toward tobacco products and companies had 1.3 to 6.3 times higher odds of greater tobacco use (both for poly-use *versus* single or non-use and for poly or single-use *versus* non-use) than students with less positive beliefs.

### 3.2. Discussion

This study indicates that a high percentage of high school students in North Carolina report using more than one tobacco product (19.1%) and that this prevalence is significantly greater than single tobacco use (10.6%). Among poly-tobacco users, cigarettes were the most frequently used in combination with another product, and the two most common combinations of tobacco products were cigarettes and cigars/cigarillos/or little cigars and cigarettes and e-cigarettes. A higher proportion of poly-users (compared to single-users and non-users) had positive beliefs regarding tobacco products and companies and beliefs were found to be significant correlates of greater tobacco use, with high school students who endorsed more positive beliefs towards tobacco companies and tobacco products more likely to report poly-use *versus* single or non-use and poly or single-use *versus* non-use.

We found that the prevalence of poly-tobacco product use among NC high school students (19.1% in 2013) was higher than findings reported by other studies, including the most recently released 2014 NYTS, which found that prevalence of using of more than one tobacco product among high school students was 12.7%. There are several reasons why poly-tobacco product use may be higher in North Carolina in our study than what has been reported nationally. First, North Carolina is a major tobacco growing state which has traditionally had higher tobacco use patterns [13]. Although North Carolina has achieved strong smoke-free legislation covering restaurants and bars, there is no comprehensive coverage in workplaces and there continues to be some form of preemption [13]. Moreover, funds from the Master Settlement Agreement in North Carolina earmarked for tobacco use prevention were transferred away amidst budget cuts to the tobacco control programs [18,19]. Rapid increases in e-cigarette use may also be associated with increasing poly-tobacco use [3]. Lastly, North Carolina has one of the lowest cigarette taxes ($0.45) compared to federal rates ($1.01) [20].

Given the high prevalence of poly-tobacco use and the higher prevalence of poly-tobacco use in comparison to single use, prevention efforts, such as communication campaigns are needed to address this new trend in tobacco use behavior. The results of this study suggest that future campaigns may expand their target audience by broadening their focus from cigarette specific prevention messages to include other types of tobacco products. Moreover, our results are consistent with previous research on the importance of beliefs as significant predictors of poly-tobacco use for traditional tobacco products [21]. Additionally, longitudinal research has found that more positive beliefs toward smoking during adolescence is associated with reduced support for smoke-free policies in adulthood, such as prohibiting smoking in bars and eliminating smoking on television and in movies [22]. The strong association between beliefs and tobacco use behavior found in our study and in other studies suggest that targeting students’ beliefs about poly-tobacco use may be a way for public health practitioners and anti-smoking communication campaigns to further reduce tobacco product use and encourage support for tobacco control policies. Perhaps media campaigns should develop and evaluate clear messages about the harmful health effects of poly-tobacco use of tobacco products rather than focusing communication campaigns on a single tobacco product like cigarettes.

Our study found that emerging tobacco products (*i.e.*, e-cigarettes) were used by a significant portion of poly-tobacco users. These findings are similar to those of other national studies, which have found that among dual and poly-tobacco users, cigarettes are commonly combined with cigars, smokeless tobacco, hookah and e-cigarettes [5,6]. Unfortunately, emerging tobacco products, such as hookah and e-cigarettes, are not currently under regulation by the Food and Drug Administration (FDA). Although the 2009 Family Smoking Prevention and Tobacco Control Act granted the FDA regulatory authority over tobacco products for the protection of public health, this does not yet include authority over emerging products, such as e-cigarettes or hookah [2,23]. In 2014, the FDA released its new “deeming rule”, which would extend its regulatory authority and some of the provisions of the Family Smoking Prevention and Tobacco Control Act to e-cigarettes, cigars, pipe tobacco, certain dissolvables that are not “smokeless tobacco”, gels, and waterpipe tobacco [24]. However, even if passed, the deeming rule may not change current advertising rules. While the Master Settlement Agreement of 1998 restricts tobacco companies from marketing products to youth, it only applies to specific products, including cigarettes and smokeless tobacco products [25,26]. Thus, despite an increase in use of potentially harmful tobacco products, the FDA currently has relatively little regulatory power over these products [2]. This raises several concerns for future tobacco control efforts, especially given that one of the most frequently used combinations of tobacco products by poly-tobacco users in our study was cigarettes and e-cigarettes (second only to cigarettes and cigars/cigarillos/or little cigars). In a diverse product market, increased regulation over conventional tobacco products (*i.e.*, cigarettes) may unintentionally encourage use of unregulated emerging products, such as e-cigarettes. Therefore, national, state, and local advocacy and policy for tobacco products currently outside the reach of FDA regulation is needed.

Increased surveillance is also needed to monitor uptake of poly-tobacco use, determine which products may be used in combination with others, determine how beliefs toward tobacco products and companies differ by type of tobacco user, and identify patterns in poly-tobacco use. Additionally, it will be important to differentiate between poly-users, single-users, and non-users when collecting and analyzing data, as our study illustrated differences in overall prevalence and beliefs for each group. Longitudinal data may be particularly useful in understanding trends in poly-tobacco use over time and establish the temporality needed to identify causal predictors of poly-tobacco use. Qualitative research will be of importance to help elucidate mechanisms leading to multiple use, potential pathways for intervening to increase prevention and control mechanisms, and how students use tobacco products.

There are several limitations of our study. First, the cross-sectional nature of the data limited our ability to make causal claims regarding the relationships between beliefs and poly use. However, our findings are consistent with known causal factors for cigarette use [9,10,11]. Second, results may not be generalizable to other students in the United States or those of other ages; however, our findings are consistent with those reported nationally [5,6]. Third, variables were assessed using students’ self-report, which could have introduced some measurement error. Fourth, four of the seven belief items in the NCYTS only asked about opinions of cigarettes (rather than tobacco products). Future research needs to understand how beliefs about novel tobacco products are related to poly-tobacco use.

## 4. Conclusions

Our study demonstrated that: (a) a high percentage of high school students in North Carolina report using more than one tobacco products concurrently, (b) positive beliefs about tobacco are significantly associated with poly-tobacco use, and (c) that emerging tobacco products are increasingly being used in combination with other tobacco products. Public health interventions and communication campaign messages focused on tobacco prevention and control may be useful in decreasing concurrent tobacco product use, especially if they target beliefs and/or poly-tobacco use of products as opposed to single tobacco product use only. Tobacco control regulation efforts are particularly important for reducing emerging tobacco product use by adolescents; increased surveillance, longitudinal research and qualitative studies to help explain experimentation, initiation and poly use of conventional and emerging tobacco products are also needed.

## Supplementary Files

Supplementary File 1
