# Supplementary material for: Poly-Tobacco Use among High School Students
Source: Int J Environ Res Public Health. 2015 Nov 13;12(11):14477–89. doi: 10.3390/ijerph121114477 (PMC4661661; doi:10.3390/ijerph121114477)
Supplement: Supplementary File 1 [file ijerph-12-14477-s001.pdf]

Poly-Tobacco Use among High School Students

Table S1. Questions Regarding Beliefs towards Tobacco Companies and Tobacco Products in the 2013 North Carolina Youth Tobacco Survey.

Question	Responses
Do you believe that tobacco companies try to get young people under 18 to use tobacco products?	A. Yes
	B. No
Do you think that breathing smoke from other people's cigarettes or other tobacco products is...?	A. Very harmful to one's health
	B. Somewhat harmful to one's health
	C. Not very harmful to one's health
	D. Not harmful at all to one's health
Do you think smoking cigarettes makes young people look cool or fit in?	A. Definitely yes
	B. Probably yes
	C. Probably not
	D. Definitely not
Do you think young people who smoke cigarettes have more friends?	A. Definitely yes
	B. Probably yes
	C. Probably not
	D. Definitely not
How strongly do you agree with the statement "All tobacco products are dangerous"?	A. Strongly agree
	B. Agree
	C. Disagree
	D. Strongly disagree
Do you think you will smoke a cigarette in the next year?	A. Definitely yes
	B. Probably yes
	C. Probably not
	D. Definitely not
If one of your best friends were to offer you a cigarette, would you smoke it?	A. Definitely yes
	B. Probably yes
	C. Probably not
	D. Definitely not
